# Giving new meaning to the impact of touch in Shantala massage: mothers’ perceptions of maternal and child well-being

**DOI:** 10.1590/0034-7167-2024-0012

**Published:** 2024-09-30

**Authors:** Danton Matheus de Souza, Letícia Sangali, Fernanda Marçal Ferreira, Samia Ahmad Ghandour, Isabelle Cristina Nogueira da Silva, Lisabelle Mariano Rossato

**Affiliations:** I Universidade de São Paulo. São Paulo, São Paulo, Brazil.

**Keywords:** Shantala, Massage, Maternal Welfare, Infant Welfare, Health Promotion, Shantala, Masaje, Bienestar Materno, Bienestar del Lactante, Promoción de la Salud

## Abstract

**Objectives:**

to understand maternal perceptions of maternal and child well-being based on Shantala massage and discuss its association with the third Sustainable Development Goal.

**Methods:**

a descriptive-exploratory study in the light of Symbolic Interactionism. Eight women, mothers of infants, participated in five online meetings to teach Shantala massage, collected in focus groups, between November and December 2021. The data was subjected to thematic content analysis and lexical analysis with IRAMUTEQ®.

**Results:**

two categories emerged, 1) Maternal perceptions of Shantala massage and its promotion of child well-being and 2) Maternal perceptions of Shantala massage and its impact on their well-being, interconnected with subcategories.

**Final Considerations:**

Shantala massage promoted mutual impacts on maternal and child well-being, working together to achieve the third Sustainable Development Goal.

## RESUMEN

**Objetivos:**

comprender las percepciones maternas sobre el bienestar materno infantil a partir del masaje Shantala y discutir su asociación con el tercer Objetivo de Desarrollo Sostenible.

**Métodos:**

estudio descriptivo-exploratorio a la luz del Interaccionismo Simbólico. Ocho mujeres, madres de bebés, participaron en cinco encuentros online para enseñar el masaje Shantala, recopilados en grupos focales, entre noviembre y diciembre de 2021. Los datos fueron sometidos a análisis de contenido temático y análisis léxico con IRAMUTEQ®.

**Resultados:**

surgieron dos categorias, 1) Percepciones maternas sobre el masaje Shantala y su promoción del bienestar infantil y 2) Percepciones maternas sobre el masaje Shantala y el impacto en su bienestar, interconectadas con subcategorias.

**Consideraciones Finales:**

el masaje Shantala promovió impactos mutuos en el bienestar materno infantil, trabajando juntos para alcanzar el tercer Objetivo de Desarrollo Sostenible.

## INTRODUCTION

Well-being promotion for all people and the security of a healthy life are one of the objectives proposed by the United Nations (UN) in the 2030 Agenda for sustainable development in the world. The third Sustainable Development Goal (SDG) deals with issues related to the population’s health, focusing on the main problems to be faced, such as preventable deaths of newborns and children under five years of age, and premature mortality from non-communicable diseases, through prevention and treatment, well-being and health promotion^(1)^.

Care approaches that encourage the bond between child and caregiver are important, combined with maternal and child well-being promotion with lifelong impacts^(2, 3)^, and the quality of this bond and attachment in parenting will be directly related to the outcomes, composing a predictor of physical health as an adult^(4, 5)^. Therefore, the adoption of strategies that contribute to establishing a positive parental bond is in line with the work developed to achieve the SDGs in the country.

Integrative and Complementary Health Practices (ICHP), established as a national policy in Brazil in 2006, have recognized the importance of promoting health and well-being through therapeutic resources, such as the *Shantala* massage technique, recognized by the policy in 2017 as one of the ICHP^(6)^. This massage was described by obstetrician Frederick Leboyer in the 1970s, being used to promote emotional bonds, trust and security in parenting as well as motor development, among other benefits for children^(7)^.

The technique consists of sliding and twisting movements, repeated five to ten times, starting with the chest and moving on to the upper, lower limbs and dorsal region, ending with gentle, circular movements on children’s face, ending with bath immersion. The relationship between children and those who apply the technique is important as is the ambiance^(7)^.

A review on the effects of physical contact points to positive results in several areas for children, influencing their socio-emotional development and bond quality, including aspects such as sleep organization, temperature and heart rate regulation, behavioral response, crying and colic^(5)^. Other studies point to the benefits of body techniques for child development, brain maturation, effects on weight gain, among other areas^(3, 8, 9)^.

It is noted that studies on touch, physical contact and massage techniques focus on newborns, mostly in a hospital setting, with a quantitative approach^(3, 5, 8, 9)^. Thus, there is a gap in the maternal perspective regarding using these home care practices beyond this age group, which may include *Shantala* massage. Considering that the premise of massage is to be performed daily, we saw it as an important part of this process to understand the perceptions and sensations generated in persons applying the technique; in this study, the maternal figure. Thus, the following research question emerged: what are the perceptions of women, mothers of infants, about performing *Shantala* massage on maternal and child well-being?

## OBJECTIVES

To understand maternal perceptions of maternal and child well-being based on *Shantala* massage and discuss its association with the third SDG.

## METHODS

### Ethical aspects

The study respected the ethical guidelines based on Resolutions 466/10 and 510/16 of the Brazilian National Health Council as well as followed the guidelines for conducting research in a virtual environment in Circular Letter 2/2021 of the Brazilian National Research Ethics Commission^(10)^. The study was approved by the *Escola de Enfermagem, Universidade de São Paulo* (EEUSP), Research Ethics Committee. The Informed Consent Form (ICF) was obtained from all individuals involved in the study online. As a way of indicating participants’ speeches, the letter M was chosen, the order of inclusion in the study and the indication of infants’ age in months: (M1; 8-month old infant) (M2; 3-month old infant).

### Theoretical-methodological framework

To better understand maternal perceptions with the use of *Shantala* massage, we chose to guide the study in light of Herbert Blumer’s Symbolic Interactionism^(11)^. This theoretical framework is based on the view that perception construction occurs through interactions in a social dynamic. To this end, individuals (mother figure) experience social interactions (with the group’s researcher guide, other mothers and their child), which generates actions (performing *Shantala* massage for five consecutive weeks). Actions are interpreted (concept of mind), generating beliefs, opinions and perceptions (concept of self), thus building symbols (in this study: perceptions about the use of *Shantala* massage for the dyad’s well-being).

### Study design

This is a descriptive-exploratory study, with a qualitative approach. For its writing, the Consolidated criteria for REporting Qualitative research (COREQ) was used^(12)^.

### Study setting

The study was conducted remotely, through five meetings with mothers on Google Meets®. This approach was chosen due to the declaration of the coronavirus disease (COVID-19) pandemic in March 2020^(13)^, which made in-person meetings in the spaces offered by the university unfeasible.

### Data source

Women over 18 years old, Brazilian or who understood Portuguese without difficulty, with internet access, with at least one infant child (between 28 days and one year old), without preexisting illnesses and who participated in the educational group meetings for *Shantala* massage, were included. Women who missed more than one meeting in a row were excluded, considering the loss of more than two stages that would prevent them from understanding the entire therapeutic resource technique.

### Data collection and organization

Participant selection was conducted using the snowball sampling technique. To this end, a folder was created to publicize the educational group for teaching *Shantala* massage, published on the social networks of the university’s research group and on the researchers’ profiles, with a request for transfer. Along with the folder, a form was published, accessed by mothers who were interested. The ICF was attached to the form, with the option of accepting the study and subsequently forwarded to filling out characterization variables. Mothers were asked if they knew any women who might be interested in the study, with a request to forward the folder and form to other mothers; this recruitment took one month (September 2021).

As mothers accepted participation, they were included in a WhatsApp® group, used as a vehicle of communication for the study, without encouraging conversations and without establishing a relationship with the researchers. Ten women were interested in the study, but two did not attend the scheduled meetings. Thus, eight women participated.

Between November and December 2021, five online meetings were held with mothers, one per week, in the evening, teaching the *Shantala* massage technique and discussing their perceptions. To teach the technique, researchers carried out a theoretical presentation, through slides, which contained the contextualization of the massage and its stages, and a practical period, with encouragement for mothers to perform the massage at that moment on their children, with observation and clarification of doubts by the researcher.

Qualitative data collection took place during the meetings, using the focus group technique, with discussions guided by the guiding phrase “Tell me how it is for you to perform *Shantala* massage”, with discourse exploration based on maternal responses. Throughout the week, the researchers sent daily reminders to encourage people to continue performing the massage. There was no pilot test. Chart 1 presents the structure of the educational groups conducted in the research.

**Chart 1:** Structuring the *Shantala* massage educational groups conducted in the research, São Paulo, São Paulo, Brazil, 2021

Meeting	Duration	Points covered
1^st^ meeting	40 minutes	Introduction of researchers, mothers and study organization; discussion about maternal expectations and fears; conceptual aspects of *Shantala* massage (concept, benefits and guidance); beginning of massage training in the thoracic region; qualitative data collection by the focus group.
2^nd^ meeting	65 minutes	Recapitulation of previous meeting, solving doubts; feedback from the previous week; follow-up of massage training with the upper limbs; qualitative data collection by the focus group.
3^rd^ meeting	50 minutes	Recapitulation of previous meeting, solving doubts; feedback from the previous week; follow-up of massage training in the abdominal region and lower limbs; qualitative data collection by the focus group.
4^th^ meeting	35 minutes	Recapitulation of previous meeting, solving doubts; feedback from the previous week; follow-up of massage training on the dorsal portion, limbs (upper and lower) and face; carrying out a complete massage; qualitative data collection by the focus group.
5^th^ meeting	35 minutes	Recapitulation of previous meetings, solving doubts; carrying out a complete massage; moment of study feedback; collection of qualitative data by the focus group; and completion of educational group.

The meetings were conducted by a researcher, nurse, professor, with training in the *Shantala* massage technique, experience in holding educational groups and conducting focus groups, together with two scientific initiation students from the EEUSP bachelor’s degree in nursing course, who provided technological assistance to the researcher as well as taking part in participant observation in the focus group.

All meetings were recorded by video, generating 225 minutes, which were transcribed in full by a researcher and validated by the main researcher. There was no discussion regarding data theoretical saturation, with collection ending for convenience at the end of the five scheduled meetings. No field notes were taken, and recordings or transcriptions were not returned to participants.

### Data analysis

The data were analyzed using Bardin’s thematic content analysis technique^(14)^, added to lexical analysis with the *Interface de R pour les Analyze Multidimensionnelles de Textes et de Questionnaires* (IRAMUTEQ)®^(15)^ and in the light of Symbolic Interactionism^(11)^.

In thematic content analysis, transcriptions were read between five and ten times (text skimming), with extraction of codes and units of meaning, grouped into theoretical categories and sub-categories^(14)^, to be described. To assist in conducting this analysis, NVivo 10 for Windows® was used. This analysis was carried out in pairs, independently, with subsequent deliberation of categorization with the other researchers, reaching a consensus. For lexical analysis using IRAMUTEQ®, a text *corpus* was prepared with only mothers’ speeches. It was decided to use the Descending Hierarchical Classification (DHC), which, through the chi-square test, allows observing the most frequent words and those with statistical differences, considered here as p<0.05 (5%), and similarity analysis, with visual representation of the connections between words in speech formation^(15)^. Lexical analysis generated 101 text segments, with a 75% success rate (considered satisfactory), with 2,280 occurrences (words, forms or vocabulary). It is noteworthy that this analysis generates classes, whereas thematic content analysis generates categories and subcategories. However, it was decided to integrate them and name them similarly (categories and subcategories).

## RESULTS

### Participant characterization

**Figure 1:**
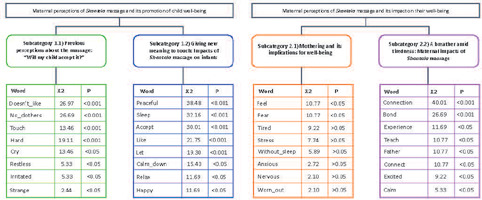
Descending Hierarchical Classification, São Paulo, São Paulo, Brazil, 2021

Eight women participated in the study, with an average age of 27 years (ranged between 23 and 33 years), all residents of the state of São Paulo, with an infant child, six of whom were males with an average age of five months (ranged between one and 11 months). Only one mother had had previous experience with *Shantala* massage, reporting that she tried to perform it on her other son, but was unsuccessful due to low acceptance. Figure 1 shows the DHC, demonstrating the categorization and the most frequent words in maternal speeches.

#### 1) Maternal perceptions of Shantala massage and its promotion of child well-being

In the subcategory “1.1) Previous perceptions about the massage: “Will my child accept it?””, there was a maternal concern regarding children’s acceptance of touch with the belief that the massage would be a challenge, considering that, in routine, children appear restless, irritable and tearful when exposed to physical manipulations, in addition to not liking being undressed (Chart 2). The three most frequent words in speeches were “doesn’t_like”, “no_clothes” and “touch” (Figure 1).

**Chart 2:** Maternal speeches in the category “Maternal perceptions of *Shantala* massage and its promotion for child well-being”, São Paulo, São Paulo, Brazil, 2021

**Subcategory 1.1 - Previous perceptions about the massage: “Will my child accept it?”**	
1^st^ meeting	*I’m scared of him* [son] *crying, because he doesn’t like being naked or being touched.* (M2; 3-month old infant) *She* [daughter] *doesn’t like me touching her, she finds it very strange, she gets restless, irritated, cries a lot.* (M6; 11-month old infant)
**Subcategory 1.2 - Giving new meaning to touch: Impacts of *Shantala* massage on infants**	
2^nd^ meeting	*I see that he* [son] *is super excited about the massage, he starts to laugh and has an impressive disposition and, in the end, he sleeps well.* (M5; 1-month old infant) *He* [son] *was very excited about the massage. He’s accepting more, he’s not finding the touch strange, it’s not bothering him as much.* (M3; 2-month old infant)
3^rd^ meeting	*After three days of repeating the massage, she* [daughter] *began to accept the massage much better, she became more peaceful, and it began to have effects.* (M8; 2-month old infant) *My son is loving the massage, I’m doing it every day before bathing to help him calm down.* (M4; 4-month old infant)
4^th^ meeting	*When he* [son] *gets irritated, massage helps him become more relaxed. Initially, it was like a joke, now he’s starting to like it more.* (M3; 2-month old infant) *I found that, for my son, it helped him to relax, he was very peaceful.* (M1; 8-month old infant)
5^th^ meeting	*He is still restless during the massage, as I’m making him start to accept more. But, although he doesn’t stay still during the massage, I see that he enjoys it, has fun, and is very excited.* (M5; 1-month old infant) *My son didn’t like being touched in some areas, like his back, but now he’s gotten used to it, he likes it, he lets it be touched.* (M3; 2-month old infant)

However, these speeches were given new meanings based on maternal interaction with *Shantala* massage, opening space for the perception of the impacts of this massage. Mothers predominantly reported that their infants relaxed during and after the massage, managing to sleep. One of the mothers reported that her son became active with the massage, wanting to play and interact. All women reported that, over time, infants became used to touch, allowing the massage to be performed and enjoying it, as seen in the subcategory “1.2) Giving new meaning to touch: Impacts of *Shantala* massage on infants” (Chart 2). In this subcategory, the most frequent words were “peaceful”, “sleep” and “accept” (Figure 1).

#### 2) Maternal perceptions of Shantala massage and its impact on their well-being

In the first meeting, mothers reflected on their well-being and mothering, reporting feeling afraid, tired, stressed, anxious and nervous. Dedication to care led to demands, sleepless nights and exhaustion, seen in the subcategory “2.1) Mothering and its implications for well-being” (Chart 3). In this subcategory, the words with statistical differences were “feel” and “fear”.

**Chart 3:** Maternal speeches in the category “Maternal perceptions of *Shantala* massage and its impact on their well-being”, São Paulo, São Paulo, Brazil, 2021

**Subcategory 2.1 - Maternal well-being? The process of caring for an infant**	
1^st^ meeting	*I’m very tired, it’s not easy to take care of a child, a first-time mother. When he becomes very restless, doesn’t sleep at night, I don’t sleep, I feel exhausted.* (M5; 1-month old infant) *We feel afraid of being a mother, it gives us anxiety, nervousness, of having to dedicate ourselves. And he* [son], *there are days when he is very angry, nothing calms down, there is no way, we get stressed.* (M7; 5-month old infant)
**Subcategory 2.2 - A breather amid tiredness: Maternal impacts of *Shantala* massage**	
2^nd^ meeting	*She* [daughter] *is sleeping well, peaceful, waking up well, not getting irritated, and this all influences me too, it reduces my anxiety, I feel calmer. My quality of life after becoming a mother is being able to sleep, and sleeping well at night helps me.* (M8; 2-month old infant)
3^rd^ meeting	*I used to be very nervous, but now I’m able to stay calmer, especially when he* [son] *is relaxed, and the massage is helping with that.* (M4; 4-month old infant) *He* [son] *is having fun with the massage; he screams and laughs a lot. I don’t see him relaxing, but rather rejoicing. And I’m happy in this process too, the whole family has fun, they come and see him during the massage, it’s attention.* (M5; 1-month old infant)
4^th^ meeting	*I’m at a stage where I’m worn out, very tired. The massage is helping with that and the meetings too, I’m becoming more willing. When he* [son] *is irritated, the massage helps him become more relaxed, and that helps me.* (M3; 2-month old infant) *I’m loving giving my daughter a massage, I feel like we’re bonding, connecting, that eye to eye.* (M6; 11-month old infant)
5^th^ meeting	*During the massage, I keep thinking, talking to him* [son]. *It ends up being a very good moment for me. I feel a greater connection with him, increasing our bond, he also becomes more connected to me.* [...] *his father wants to try doing it, I’m teaching him. I think it’s wonderful, so that he can also create that connection.* (M5; 1-month old infant)

Performing the massage on the son provided mutual impacts. Mothers reported greater “connection” and “bond” with infants, the two most frequent words, with greater excitement, reduced anxiety and nervousness, being able to sleep and reporting improved quality of life. Furthermore, they reported that the group allowed them to teach their partners so that they could also experience the impacts. The moment of massage leads to maternal pleasure and that of her extended family, as shown in the subcategory “2.2) A breather amid tiredness: Maternal impacts of *Shantala* massage” (Chart 3).

Mothers assessed the experience in the educational group and *Shantala* massage as a good experience, and the fact that it was remote helped in the possibility of participation and in encouraging continuity of the massage at home, as seen in speeches below:

*For me, this online meeting was great, because I live far away, I can organize myself. In person, I wouldn’t be able to go.* (M8; 2-month old infant)

*If it were in person, I wouldn’t be able to participate, I wouldn’t be able to reconcile.* (E3; 2-month old infant)

*I think the weekly online group was great for encouraging me to continue doing.* (E5; 1-month old infant)

**Figure 2:**
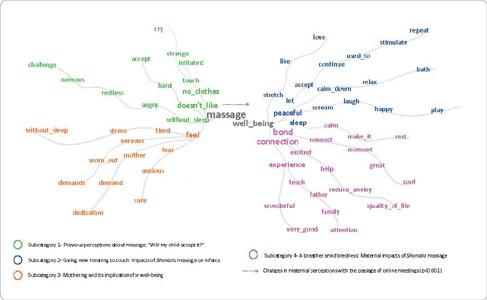
Similarity tree, São Paulo, São Paulo, Brazil, 2021

Figure 2 shows the similarity tree.

## DISCUSSION

The results of this study demonstrated that *Shantala* massage, as a therapeutic resource to promote maternal and child well-being, was given new meaning by mothers as they allowed themselves to perform the technique, guided by online educational activities, as illustrated in Figures 1 and 2, anchored by the perspective of Symbolic Interactionism. This reference considers that there is an expansion of the meanings attributed based on the way people act and interact in relation to the object^(11)^. It is noted that the change in mothers’ perceptions was the result of their interaction with the therapeutic technique and observation of infants’ acceptance of that care.

Symbolic Interactionism demonstrates that the interpretation of phenomena is not fixed, but rather dynamic, capable of being modified by actions^(11)^. This aspect is in agreement with this study. To illustrate the above, one participant had previous contact with therapeutic massage and, even so, had a negative experience, which reinforces that the interaction allowed the redefinition, both of this participant’s previous experience, when she applied the technique to her first child, and of the other participants in relation to their preconception regarding massage.

Infants’ discomfort with touching and nudity was one of the major concerns expressed in mothers’ perceptions. Although tactile stimulation is recommended to reduce stress, pain intensity, heart and respiratory rate, and improved sleep^(16)^, touch was perceived by mothers as uncomfortable for infants during the first contact with massage. In this regard, the presentation of tactile practices that seek to promote relaxation, such as *Shantala,* should be applied gradually to infants for better adaptation and acceptance^(16)^. Here five meetings were held, and it can be seen that adaptation and acceptance were quick, as women’s perceptions regarding the practice and their children’s well-being changed in the second week of online activity, following the premises of Symbolic Interactionism^(11)^, presented in Charts 2 and 3.

It is important to emphasize that, during *Shantala* practice, infants participate in the therapy as a subject of action, and not just as a passive participant in care. Therefore, each child has their preferences and discomforts, which must be respected, because, after all, the aim of massage is relaxation, not to stress infants with practices that are not well accepted. This interaction provides an opportunity for bonding between mother and child, learning to observe, communicate, understand and respect their individuality.

The stimuli and interactions received by children are of great importance for brain, emotional and intellectual development, and have an influence mainly during the prenatal period and the first three years of life^(17)^. All infants who received the massage were within the first 1,000 days of life, a period recognized for its importance and influence on this development, with long-term impacts^(18)^. Although the greatest emphasis during this period is related to adequate nutrition and affection, other care must be taken into account.

Hence, physical touch is marked as a possibility, being essential to establish positive parenting between caregivers and children^(18)^. The interaction provided by *Shantala* can be an aspect that promotes development, especially by integrating the need for continuous supportive relationships^(17)^, since, as meetings took place, children accepted the massage more and mothers became more confident. By following the technique throughout children’s growth, safe interactions can be promoted.

Knowing the repercussions that the 1,000 days will have throughout life, the importance of bonding and affection during this period for health, and considering that promoting well-being is a goal for sustainable development in the world, it is notable that actions that contribute to establishing the bond, that aim to promote health and protect maternal and child health are disseminated and implemented, which demonstrates, once again, the importance of this study in this context.

It is recommended that, at the end of the massage, children be offered a bath, a practice that contributes to continuation of relaxation^(7)^, since immersion bath in water reduces the response of the autonomic nervous system, prevailing the response of the parasympathetic nervous system. Furthermore, the memory of the intrauterine environment is mentioned as a subjective aspect that can refer to the feeling of security for infants^(19)^. The sum of the two interventions can enhance the interaction between the dyad.

Along the way, special attention was focused on the bond between mother and infant to promote joint well-being. However, there are adverse experiences that can impact this context, such as maternal mental health problems. A study on maternal anxiety in the postpartum period and its effects on breastfeeding, which also involves touch, shows a high prevalence of the disorder, with intrusive thoughts, feelings of guilt, muscle tension, among other symptoms that can make interaction with infants difficult and reduce satisfaction with maternal functions in the postpartum period^(20)^.

Another point to consider is that the meetings took place during the COVID-19 pandemic. Although no participant mentioned the influence of this scenario on their mothering experiences, it is known that, intrinsically, the impacts of social distancing measures can influence maternal mental health and their bond with their children. This was seen in a Brazilian study, in which a high prevalence of postpartum depression was observed in women during the COVID-19 pandemic, and this outcome was associated with impaired bonding with their children^(21)^.

In this study, in addition to changes in maternal perception about child well-being after practicing *Shantala,* mothers reported changes in perceptions about their own well-being as the practice of massage was learned and practiced, as seen in Figure 2, which shows the transition of sensations “anxious”, “without_sleep” and “worn_out” for “connected”, “calm” and “reduce_anxiety”. Therefore, it is worth reflecting in future studies on the potential of the intervention to reduce the aforementioned mental health problems, considering this transition to motherhood where breathing is possible, as seen in Chart 3.

This greater connection and bond with their children, reported even by those who felt distanced from their children, supports findings in the literature that describe this change in the relationship with their children^(22)^. The bond facilitates the perception of infants’ preferences during the massage, allowing time and attention to be dedicated to the parts of the body where infants express signs of satisfaction and to discontinue the practice at signs of restlessness and irritability, being a moment in which mothers can also know and learn to respect the individualities of their children^(22)^.

Another point to be highlighted is that the gradual presentation of techniques that involve tactile stimulation can facilitate not only infants’ acceptance, but also caregivers’ learning, avoiding greater concerns with the step-by-step process to be followed initially, paying attention to their children’s reactions. In an interactionist perspective, the meanings of a phenomenon are understood after reflection and interpretation^(11)^. Thus, the fact that the focus group was carried out at five different times is a potentiality of this study, as it allows women’s perceptions not to be immediate, but rather reported after a reflective process, with more than one exposure and encouragement to perform the massage. This time helps to minimize perceptions, such as that described by a study participant, of self-blame for the baby’s discomfort when touched.

The Evidence Map of the Clinical Effectiveness of *Shantala* Practice, which brought together 37 studies on this therapeutic resource, demonstrated a positive effect of the technique on the outcome “well-being, quality of life and vitality”, with an impact on growth, stress, neuropsychomotor development, sleep quality and improvement in proprioception^(23)^, which supports the maternal perceptions seen in this study. Furthermore, no data was found on the map regarding maternal well-being^(23)^. Considering this, this study was concerned with maternal perceptions about their well-being during the massage, demonstrating positive aspects and contributing to the advancement of knowledge about *Shantala* massage (Chart 3).

In online groups, mothers paid more attention to children, even being encouraged to talk about their experience. This aspect may be related to the social role of women as mothers, a phenomenon that indicates that they give themselves to the role, giving up their individuality, needs and desires, and cannot become frustrated^(24)^. Thus, the opportunity to bring together women experiencing similar life moments may have facilitated the opening for a frank conversation, resulting in the exposure of their fears and dissatisfactions with maternal functions, but being able to give new meaning to them after interacting with the massage. Supporting these findings, a Norwegian study concluded that time with children in *Shantala* massage provided relief from maternal guilt related to not constantly feeling love in their relationship with their children, which made them feel ashamed^(22)^.

Here, we focused on maternal perception. However, for future studies, it is recommended that other family members be approached to promote the benefits mentioned for everyone involved in the care process. There is an intense movement in clinical practice and scientific literature towards the inclusion of the father figure in interventions^(25)^. In this study, a mother reported that she taught the technique to the child’s father, a positive aspect that should be reproduced in future studies, to guarantee positive parenting between the dyad (mother and father).

Another aspect is that mothers perceived the online intervention as something positive and promoting their participation. After the COVID-19 pandemic, professionals began their approach to remote care^(26)^, and this study demonstrates that this organization holds promise for teaching care interventions. Furthermore, it is recommended for future studies to test the intervention in places such as primary (childcare consultations and prenatal groups) and tertiary services (maternity wards and neonatal care units), in order to enable the effects mentioned here to be experienced by children and mothers in different contexts.

In an interactionist light, meanings arise from the interaction that human beings have with others^(11)^. It can be considered that an important factor for this process, in this study, is related to the educational activities carried out collectively, supporting the findings of a study on the group care approach to maternal and child health, developed in a Central American country, which demonstrated that collective care activities allowed participants to make positive changes in health behavior for themselves and their children^(27)^.

Transition to motherhood encompasses physiological changes (hormonal and neurobiological) and in the social and environmental context, which can cause cognitive impacts, with consequences that are still uncertain and unknown until aging. Here, what is worth discussing is that the cognitive load that parenthood imposes on caregivers, mostly a mother, is so intense that it requires attention and continuous care^(28)^. Thus, as well as for child well-being, it is essential to reflect on strategies to promote maternal well-being and health to meet the 2030 Agenda goal. *Shantala* can be a simple, accessible and powerful resource for this purpose. It is worth highlighting the importance that healthcare professionals, especially nurses, have in achieving the SDGs, directing actions that promote health and well-being^(29)^.

It is reflected that, in the Brazilian scenario, although ICHP have been recognized in policy since 2006^(6)^, their insertion into professional practice is occurring gradually. For this process to accelerate and practices to be recognized as care technologies, healthcare professionals’ acceptance is necessary, with the dissemination of the benefits of this practice in studies being a starting point for an implementation process. The role of nurses in this process stands out, as seen in this study, where professionals in the area made it possible to learn *Shantala* massage online, providing benefits to maternal and child well-being, supporting the literature, which highlights the importance of this professional in dissemination of ICHP^(30)^.

### Study limitations

This study has limitations on the number of participants; however, this may be a reflection of the perceptions seen in subcategory 1.1, in which mothers were afraid to start the massage due to infants’ behavior regarding touch. This aspect reinforces the need to publish this manuscript to give new meaning to this context. The online approach, despite maternal validation as a potential for their participation, may have limited exposure of perceptions, in addition to lack of control, whether mothers conducted massages daily, despite daily sending of reminders, which could influence perceptions. Another limitation refers to all mothers being residents of a single Brazilian state, recommending that future studies work in other states and countries.

### Contributions to nursing, health and public policies

It is expected that this study will contribute to nursing based on maternal validation of a care technology (*Shantala* massage) that should be disseminated within the professional scope due to its effects on maternal and child well-being, advancing the journey towards reaching the 3^rd^ SDG.

## FINAL CONSIDERATIONS

It was observed that mothers perceived *Shantala* massage as a care that promotes mutual impacts on maternal and child well-being, being consistent with the proposal of the third SDG and allowing a path towards achieving it. Initially, mothers were afraid of performing the massage due to their child’s daily behavior and reported exhaustion with mothering. However, in their interaction with *Shantala* massage, these perceptions were given new meaning.
