# Supplementary material for: Giving new meaning to the impact of touch in Shantala massage: mothers’ perceptions of maternal and child well-being
Source: Rev Bras Enferm. 2024 Sep 30;77(Suppl 2):e20240012. doi: 10.1590/0034-7167-2024-0012 (PMC11441756; doi:10.1590/0034-7167-2024-0012)
Supplement: Translation

# Translation: Ressignificando o impacto do toque na massagem Shantala: percepções de mães sobre o bem-estar materno-infantil

**DOI:** 10.1590/0034-7167-2024-0012pt

**Authors:** Danton Matheus de Souza, Letícia Sangali, Fernanda Marçal Ferreira, Samia Ahmad Ghandour, Isabelle Cristina Nogueira da Silva, Lisabelle Mariano Rossato

**Affiliations:** I Universidade de São Paulo. São Paulo, São Paulo, Brasil.

**Keywords:** Shantala, Massagem, Bem-Estar Materno, Bem-Estar do Lactente, Promoção da Saúde

## Abstract

**Objetivos:**

compreender as percepções maternas sobre o bem-estar materno-infantil a partir da realização da massagem *Shantala* e discutir sua associação com o terceiro Objetivo do Desenvolvimento Sustentável.

**Métodos:**

estudo descritivo-exploratório à luz do Interacionismo Simbólico. Contou-se com oito mulheres, mães de lactentes, que participaram de cinco encontros *online* para o ensino da massagem *Shantala,* com coleta em grupos focais, entre novembro e dezembro de 2021. Os dados foram submetidos à análise temática de conteúdo e à análise lexical com o IRAMUTEQ®.

**Resultados:**

emergiram duas categorias, 1) Percepções maternas da massagem Shantala e sua promoção para o bem-estar infantil e 2) Percepções maternas da massagem *Shantala* e o impacto no seu bem-estar, interligadas com subcategorias.

**Considerações Finais:**

a massagem *Shantala* promoveu impactos mútuos no bem-estar materno infantil, articulando-se no caminhar para atingir o terceiro Objetivo do Desenvolvimento Sustentável.

## INTRODUÇÃO

A promoção do bem-estar para todas as pessoas e a seguridade de uma vida saudável compõem um dos objetivos propostos pela Organização das Nações Unidas (ONU) na Agenda 2030, para o desenvolvimento sustentável no mundo. O terceiro Objetivo do Desenvolvimento Sustentável (ODS) trata de questões relacionadas à saúde da população, com foco nos principais agravos a serem enfrentados, tais como as mortes evitáveis de recém-nascidos e crianças menores de cinco anos, e a mortalidade prematura por doenças não transmissíveis, através da prevenção e tratamento, promoção de bem-estar e saúde^(1)^.

Abordagens de cuidado que incentivam o vínculo entre a criança e o cuidador são importantes, aliadas para a promoção do bem-estar materno-infantil com impactos por toda a vida^(2, 3)^, sendo que a qualidade desse vínculo e de apego na parentalidade estará diretamente relacionada aos desfechos, compondo um preditor da saúde física quando adulto^(4, 5)^. Assim, a adoção de estratégias que contribuam para o estabelecimento de vínculo parental positivo consoa com o trabalho desenvolvido para alcançar os ODS no país.

As Práticas Integrativas e Complementares em Saúde (PICS), instituídas como política nacional no Brasil em 2006, têm reconhecido a importância da promoção de saúde e bem-estar por intermédio de recursos terapêuticos, como a técnica de massagem *Shantala,* reconhecida pela política em 2017 como uma das PICS^(6)^. Essa massagem foi descrita pelo médico obstetra Frédérick Leboyer na década de 70, sendo utilizada para promoção de vínculo afetivo, confiança e segurança na parentalidade, além de desenvolvimento motor, entre outros benefícios para a criança^(7)^.

A técnica consiste em movimentos de deslizamento e torções, repetidos de cinco a dez vezes, iniciando-se pelo tórax e seguindo para membros superiores, inferiores e região dorsal, com finalização em movimentos suaves e circulares na face da criança, com término em um banho de imersão. A relação entre a criança e quem aplica a técnica é importante, assim como a ambiência^(7)^.

Revisão sobre os efeitos do contato físico aponta resultados positivos em vários domínios para crianças, influenciando seu desenvolvimento socioemocional e a qualidade do vínculo, incluindo aspectos como organização do sono, regulação da temperatura e da frequência cardíaca, resposta comportamental, choro e cólica^(5)^. Outros estudos apontam sobre os benefícios das técnicas corporais para o desenvolvimento infantil, maturação cerebral, efeitos sobre ganho de peso, entre outros domínios^(3, 8, 9)^.

Nota-se que o foco dos estudos sobre técnicas de toque, contato físico e massagem é o recém-nascido, majoritariamente em contexto hospitalar, com abordagem quantitativa^(3, 5, 8, 9)^. Nesse sentido, observa-se uma lacuna no olhar sobre a perspectiva materna quanto à utilização dessas práticas de cuidado em domicílio para além dessa faixa etária, podendo-se incluir a massagem *Shantala.* Considerando que a premissa da massagem é ser realizada cotidianamente, vimos como parte importante desse processo compreender as percepções e sensações geradas na pessoa que aplica a técnica; neste estudo, a figura materna. Assim, emergiu a seguinte pergunta de pesquisa: quais são as percepções de mulheres, mães de lactentes, sobre a realização da massagem *Shantala* no bem-estar materno-infantil?

## OBJETIVOS

Compreender as percepções maternas sobre o bem-estar materno-infantil a partir da realização da massagem *Shantala* e discutir sua associação com o terceiro ODS.

## MÉTODOS

### Aspectos éticos

O estudo respeitou as diretrizes éticas pautadas nas Resoluções nº 466/10 e nº 510/16 do Conselho Nacional de Saúde, bem como seguiu as orientações para a condução de pesquisas em ambiente virtual da do Ofício Circular nº 2/2021 da Comissão Nacional de Ética em Pesquisa^(10)^. O estudo foi aprovado pelo Comitê de Ética em Pesquisa da Escola de Enfermagem da Universidade de São Paulo (EEUSP). O Termo de Consentimento Livre e Esclarecido (TCLE) foi obtido de todos os indivíduos envolvidos no estudo por meio *online.* Como forma de indicar os discursos das participantes, optou-se pela letra M, a ordem de inclusão no estudo e a indicação da idade do lactente em meses: (M1; lactente de 8 meses) (M2; lactente de 3 meses).

### Referencial teórico-metodológico

Para maior compreensão das percepções maternas com o uso da massagem *Shantala,* optou-se por guiar o estudo à luz do Interacionismo Simbólico de Herbert Blumer^(11)^. Esse referencial teórico pauta-se na visão de que a construção de percepções ocorre por intermédio das interações em uma dinâmica social. Para tal, o indivíduo (figura materna) vivencia interações sociais (com o pesquisador guia do grupo, outras mães e seu filho), o que gera ações (realização da massagem *Shantala* por cinco semanas seguidas). As ações são interpretadas (conceito de mente), gerando as crenças, opiniões e percepções (conceito de *self*), construindo, assim, os símbolos (neste estudo: as percepções sobre o uso da massagem *Shantala* para o bem-estar do binômio).

### Tipo de estudo

Trata-se de estudo descritivo-exploratório, de abordagem qualitativa. Para a sua redação, utilizou-se o instrumento *Consolidated criteria for REporting Qualitative research* (COREQ)^(12)^.

### Cenário do estudo

O estudo foi conduzido de forma remota, por intermédio de cinco encontros com as mães na plataforma *Google Meets*®. Essa abordagem foi escolhida devido à declaração da pandemia da doença do coronavírus (COVID-19) em março de 2020^(13)^, o que inviabilizou os encontros presenciais nos espaços ofertados pela universidade.

### Fonte de dados

Foram incluídas mulheres, acima de 18 anos, brasileiras ou que compreendessem a língua portuguesa sem dificuldades, com acesso à internet, com no mínimo um filho lactente (compreendido entre 28 dias e um ano incompleto), sem doenças preexistentes e que participassem dos encontros do grupo educativo para a massagem *Shantala.* Foram excluídas as mulheres que faltaram em mais de um encontro seguido, considerando a perda de mais de duas etapas que as impediriam de compreender toda a técnica do recurso terapêutico.

### Coleta e organização dos dados

A seleção dos participantes foi conduzida pela técnica de amostragem bola de neve. Para tal, foi realizado um *folder* de divulgação do grupo educativo para ensino da massagem *Shantala,* divulgado nas redes sociais do grupo de pesquisa da universidade e nos perfis dos pesquisadores, sendo solicitado um repasse. Junto ao *folder,* era divulgado um formulário, acessado pelas mães que apresentavam interesse. Nesse, era anexado o TCLE, com a opção do aceite ao estudo e posteriormente encaminhado a um preenchimento de variáveis de caracterização. As mães eram questionadas se não conheciam mulheres que poderiam se interessar pelo estudo, com solicitação do encaminhamento do *folder* e formulário às outras mães; esse percurso de recrutamento levou um mês (setembro de 2021).

Conforme as mães aceitavam a participação, eram incluídas em um grupo de *WhatsApp*®, utilizado como um veículo de comunicação para o estudo, sem estímulo a conversas e sem estabelecimento de relacionamento com os pesquisadores. Dez mulheres apresentaram interesse no estudo, porém duas não foram aos encontros agendados. Assim, oito mulheres participaram.

Entre novembro e dezembro de 2021, foram conduzidos cinco encontros *online* com as mães, um por semana, no período noturno, com o ensino da técnica de massagem *Shantala* e discussão quanto às suas percepções. Para o ensino da técnica, os pesquisadores realizaram uma exposição teórica, por meio de *slides,* que continha a contextualização da massagem e suas etapas, e um período prático, com o estímulo para que as mães realizassem a massagem naquele momento em seus filhos, com observação e retirada de dúvidas pela pesquisadora.

A coleta de dados qualitativos ocorria durante a realização dos encontros, com a técnica de grupo focal, com discussões guiadas pela frase norteadora “Conte-me como está sendo para você realizar a massagem *Shantala”*, com exploração do discurso a partir das respostas maternas. Ao longo da semana, os pesquisadores encaminhavam lembretes diários para o estímulo da continuidade da realização da massagem. Não houve teste piloto. O Quadro 1 apresenta a estruturação dos grupos educativos conduzidos na pesquisa.

**Quadro 1:** Estruturação dos grupos educativos de massagem *Shantala* conduzidos na pesquisa, São Paulo, São Paulo, Brasil, 2021

Encontro	Duração	Pontos abordados
1º encontro	40 minutos	Apresentação dos pesquisadores, das mães e da organização do estudo; discussão sobre as expectativas e medos maternos; aspectos conceituais da massagem *Shantala* (conceito, benefícios e condução); início do treinamento da massagem pela região torácica; coleta de dados qualitativos pelo grupo focal.
2º encontro	65 minutos	Recapitulação do encontro anterior, com retirada de dúvidas; *feedback* da semana anterior; seguimento do treinamento da massagem com os membros superiores; coleta de dados qualitativos pelo grupo focal.
3º encontro	50 minutos	Recapitulação do encontro anterior, com retirada de dúvidas; *feedback* da semana anterior; seguimento do treinamento da massagem na região abdominal e membros inferiores; coleta de dados qualitativos pelo grupo focal.
4º encontro	35 minutos	Recapitulação do encontro anterior, com retirada de dúvidas; *feedback* da semana anterior; seguimento do treinamento da massagem na porção dorsal, membros (superiores e inferiores) e face; realização da massagem completa; coleta de dados qualitativos pelo grupo focal.
5º encontro	35 minutos	Recapitulação dos encontros anteriores, com retirada de dúvidas; realização da massagem completa; momento de *feedback* do estudo; coleta de dados qualitativos pelo grupo focal; e finalização do grupo educativo.

Os encontros foram conduzidos por uma pesquisadora, enfermeira, professora universitária, com formação na técnica de massagem *Shantala,* experiência na realização de grupos educativos e condução de grupos focais, em conjunto com duas discentes de iniciação científica do curso de bacharelado em enfermagem da EEUSP, que forneciam auxílio tecnológico à pesquisadora, bem como faziam parte da observação participante no grupo focal.

Todos os encontros foram gravados por vídeo, gerando 225 minutos, que foram transcritos na íntegra por um pesquisador e validado pela pesquisadora principal. Não houve discussão quanto à saturação teórica dos dados, com a finalização da coleta por conveniência, ao término dos cinco encontros agendados. Não foram feitas notas de campo, e as gravações ou transcrições não foram devolvidas às participantes.

### Análise dos dados

Os dados foram analisados pela técnica de análise temática de conteúdo de Bardin^(14)^, somada à análise lexical com o *software Interface de R pour les Analyse Multidimensionnelles de Textes et de Questionnaires* (IRAMUTEQ)®^(15)^ e à luz do Interacionismo Simbólico^(11)^.

Na análise temática de conteúdo, as transcrições foram lidas entre cinco e dez vezes (leitura flutuante), com extração de códigos e unidades de significado, agrupados em categorias teóricas e subcategorias^(14)^, a serem descritas. Para o auxílio na condução desta análise, utilizou-se o *software NVivo 10 for Windows*®. Essa análise foi realizada em pares, de forma independente, com posterior deliberação da categorização aos demais pesquisadores, chegando a um consenso. Para a análise lexical pelo IRAMUTEQ®, foi preparado um *corpus* textual apenas com os discursos maternos. Optou-se por utilizar a Classificação Hierárquica Descendente (CHD), que, por meio do teste qui-quadrado, permite observar as palavras mais frequentes e aquelas com diferença estatística, aqui considerada como o p<0,05 (5%), e a análise da similitude, com a representação visual das conexões entre as palavras na formação do discurso^(15)^. A análise lexical gerou 101 segmentos de texto, com aproveitamento de 75% (considerado satisfatório), com 2.280 ocorrências (palavras, formas ou vocabulários). É digno de nota que essa análise gera classes, enquanto que a análise temática de conteúdo gera categorias e subcategorias. Contudo, optou-se por integrá-las e denominá-las de forma semelhante (categorias e subcategorias).

## RESULTADOS

### Caracterização dos participantes

Participaram do estudo oito mulheres, com média de idade de 27 anos (variou entre 23 e 33 anos), todas residentes do estado de São Paulo, com um filho lactente, sendo seis do sexo masculino com idade média de cinco meses (variou entre um e 11 meses). Apenas uma mãe já tinha tido experiência prévia com a massagem *Shantala,* com relato de ter tentado realizar em seu outro filho, mas não obteve sucesso, pela baixa aceitação. A Figura 1 traz a CHD, demonstrando a categorização e as palavras mais frequentes nos discursos maternos.

**Figura 1:** Classificação Hierárquica Descendente, São Paulo, São Paulo, Brasil, 2021

#### 1) Percepções maternas da massagem Shantala e sua promoção para o bem-estar infantil

Na subcategoria “1.1) Percepções prévias sobre a massagem: “Será que meu filho vai aceitar?””, observou-se uma preocupação materna diante da aceitação do filho ao toque com a crença de que a massagem seria um desafio, considerando que, na rotina, o filho se mostra agitado, irritado e choroso às manipulações físicas, somado a não gostar de ficar despido (Quadro 2). As três palavras mais frequentes nos discursos foram “não_gosta”, “sem_roupa” e “toque” (Figura 1).

**Quadro 2:** Discursos maternos diante da categoria “Percepções maternas da massagem *Shantala* e sua promoção para o bem-estar infantil”, São Paulo, São Paulo, Brasil, 2021

**Subcategoria 1.1 - Percepções prévias sobre a massagem: “Será que meu filho vai aceitar?”**	
1º encontro	*Eu fico com medo dele* [filho] *chorar, porque ele não gosta de ficar sem roupa e nem de ser tocado.* (M2; lactente de 3 meses) *Ela* [filha] *não gosta que eu toque nela, estranha muito o toque, fica agitada, irritada, chora demais.* (M6; lactente de 11 meses)
**Subcategoria 1.2 - Ressignificando o toque: Impactos da massagem *Shantala* nos lactentes**	
2º encontro	*Eu vejo que ele* [filho] *fica superanimado com a massagem, ele começa a rir e fica com uma disposição impressionante e, ao final, ele dorme bem.* (M5; lactente de 1 mês) *Ele* [filho] *ficou bem animado com a massagem. Ele está aceitando mais, não está estranhando o toque, não está incomodando tanto.* (M3; lactente de 2 meses)
3º encontro	*Depois de três dias repetindo a massagem, ela* [filha] *começou a aceitar a massagem muito melhor, ficou mais tranquila, começou a ter efeitos.* (M8; lactente de 2 meses) *Meu filho está amando a massagem, eu estou fazendo todo o dia antes do banho para ajudar ele a se acalmar.* (M4; lactente de 4 meses)
4º encontro	*Quando ele* [filho] *fica irritado, a massagem ajuda a ficar mais relaxado. Inicialmente, era como se fosse uma brincadeira, agora ele está começando a gostar mais.* (M3; lactente de 2 meses) *Eu achei que, pro meu filho, ajudou ele a relaxar, ele ficou bem tranquilo.* (M1; lactente de 8 meses)
5º encontro	*Ele ainda fica agitado durante a realização da massagem, conforme estou fazendo ele começa a aceitar mais. Mas, apesar de ele não ficar quieto na massagem, eu vejo que ele gosta, se diverte, fica muito empolgado.* (M5; lactente de 1 mês) *O meu filho não gostava do toque em algumas regiões, como nas costas, mas agora ele já se acostumou, gosta, deixa o toque ser realizado.* (M3; lactente de 2 meses)

Contudo, esses discursos foram ressignificados a partir da interação materna com a realização da massagem *Shantala,* abrindo espaço para a percepção dos impactos da massagem. As mães relataram, prevalentemente, que os lactentes relaxavam durante e após a massagem, conseguindo dormir. Uma das mães relatou que seu filho ficava ativo com a massagem, querendo brincar e interagir. Todas as mulheres referiram que, com o passar do tempo, o lactente se acostumou com o toque, permitindo a realização da massagem e gostando, conforme visto na subcategoria “1.2) Ressignificando o toque: Impactos da massagem *Shantala* nos lactentes” (Quadro 2). Nessa subcategoria, as palavras mais frequentes foram “tranquilo”, “dormir” e “aceitar” (Figura 1).

#### 2) Percepções maternas da massagem Shantala e o impacto no seu bem-estar

No primeiro encontro, as mães refletiram quanto ao seu bem-estar e à maternagem, referindo sentir medo, cansaço, estresse, ansiedade e nervosismo. A dedicação ao cuidado levou a cobranças, noites sem dormir e esgotamento, visto na subcategoria “2.1) Maternagem e suas implicações ao bem-estar” (Quadro 3). Nessa subcategoria, as palavras com diferença estatística foram “sentir” e “medo”.

**Quadro 3:** Discursos maternos diante da categoria “Percepções maternas da massagem *Shantala* e o impacto no seu bem-estar”, São Paulo, São Paulo, Brasil, 2021

**Subcategoria 2.1 - Bem-estar materno? O processo de cuidar de um lactente**	
1º encontro	*Eu estou bem cansada, não é fácil cuidar de um filho, mãe de primeira viagem. Quando ele fica muito agitado, não dorme à noite, eu fico sem dormir, fico esgotada.* (M5; lactente de 1 mês) *A gente sente medo de ser mãe, dá uma ansiedade, um nervosismo, de ter que se dedicar. E ele* [filho], *tem dias que está muito bravo, que nada acalma, não tem como, ficamos estressadas.* (M7; lactente de 5 meses)
**Subcategoria 2.2 - Um respiro em meio ao cansaço: Impactos maternos da massagem *Shantala***	
2º encontro	*Ela* [filha] *está dormindo bem, tranquila, acordando bem, não ficando irritada, e isso tudo influencia em mim também, reduz minha ansiedade, eu fico mais calma. Minha qualidade de vida depois de me tornar mãe é poder dormir, e ela dormir bem à noite me ajuda.* (M8; lactente de 2 meses)
3º encontro	*Já tive época de ficar muito nervosa, agora estou conseguindo ficar mais calma, principalmente quando ele* [filho] *está relaxado, e a massagem está ajudando nisso.* (M4; lactente de 4 meses) *Ele* [filho] *está se divertindo com a massagem, ele grita e dá muita risada. Não vejo ele relaxando, e sim se alegrando. E eu fico alegre nesse processo também, a família toda se diverte, vem ver ele na hora da massagem, é uma atenção.* (M5; lactente de 1 mês)
4º encontro	*Eu estou em uma fase que estou esgotada, muito cansada. A massagem está ajudando nisso e os encontros também, estou ficando mais disposta. Quando ele* [filho] *está irritado, a massagem vai ajudando para ele ficar mais relaxado, e isso me ajuda.* (M3; lactente de 2 meses) *Eu estou amando fazer a massagem na minha filha, sinto que estamos nos vinculando, nos conectando, aquele olho no olho.* (M6; lactente de 11 meses)
5º encontro	*Durante a massagem, eu fico mentalizando, conversando com ele* [filho]. *Acaba que é um momento muito bom para mim. Eu sinto uma conexão maior com ele, aumentando nosso vínculo, ele também fica mais conectado comigo.* [...] *o pai dele quer tentar fazer, eu estou ensinando. Acho que é maravilhoso, para que ele também possa criar essa conexão.* (M5; lactente de 1 mês)

A realização da massagem no filho proporcionou impactos mútuos. As mães referiram maior “conexão” e “vínculo” com o lactente, sendo as duas palavras mais frequentes, com maior empolgação, redução da ansiedade e nervosismo, conseguindo dormir e referindo melhorar a sua qualidade de vida. Ainda, relataram que o grupo permitiu que elas ensinassem aos parceiros, para que também vivenciassem os impactos. O momento da massagem leva ao prazer materno e à sua família extensa, como mostra a subcategoria “2.2) Um respiro em meio ao cansaço: Impactos maternos da massagem *Shantala”* (Quadro 3).

As mães avaliaram a experiência no grupo educativo e a condução da massagem *Shantala* como uma boa experiência, sendo que o fato de ser remoto auxiliou na possibilidade da participação e no estímulo à continuidade da realização em domicílio, como visto nos discursos abaixo:

*Para mim, esse encontro online foi ótimo, porque eu moro longe, consigo me organizar. Presencial, eu não teria condição de ir.* (M8; lactente de 2 meses)

*Se fosse presencial, eu não conseguiria participar, não conseguiria conciliar.* (E3; lactente de 2 meses)

*Eu acho que o grupo online, semanalmente, foi ótimo para me estimular a continuar fazendo.* (E5; lactente de 1 mês)

A Figura 2 traz a árvore da similitude.

**Figura 2:** Árvore da similitude, São Paulo, São Paulo, Brasil, 2021

## DISCUSSÃO

Os resultados deste estudo demonstraram que a massagem *Shantala,* como um recurso terapêutico para promoção de bem-estar materno-infantil, foi ressignificada pelas mães conforme permitiram-se realizar a técnica, guiadas a partir das atividades educativas *online,* como ilustrado nas Figuras 1 e 2, ancorados pela perspectiva do Interacionismo Simbólico. Esse referencial considera que há expansão dos significados atribuídos a partir da forma como as pessoas agem e interagem em relação ao objeto^(11)^. Nota-se que a mudança das percepções das mães foi resultado da sua interação com a técnica terapêutica e a observação da aceitação daquele cuidado pelos lactentes.

O Interacionismo Simbólico demonstra que a interpretação dos fenômenos não é fixa, mas sim dinâmica, passível de ser modificada por ações^(11)^. Esse aspecto está de acordo com este estudo. Para ilustração do citado, uma participante tinha contato prévio com a massagem terapêutica e, ainda assim, com experiência negativa, o que reforça que a interação permitiu a ressignificação, tanto da vivência anterior dessa participante, quando aplicara a técnica em seu primeiro filho, quanto das demais participantes em relação ao pré-conceito em relação à massagem.

O desconforto dos lactentes diante do toque e da nudez foi uma das grandes preocupações manifestadas nas percepções das mães. Embora a estimulação tátil seja recomendada para reduzir o estresse, a intensidade da dor, a frequência cardíaca e respiratória, e a melhora do sono^(16)^, o toque foi percebido pelas mães como desconfortável para o lactente no primeiro contato com a massagem. Nesse sentido, a apresentação de práticas táteis que buscam promover o relaxamento, como a *Shantala,* deve ser aplicada de forma gradual aos lactentes para melhor adaptação e aceitação^(16)^. Aqui, foram conduzidos cinco encontros, e nota-se que a adaptação e a aceitação foram rápidas, visto que as percepções das mulheres em relação à prática e ao bem-estar de seus filhos mudaram já na segunda semana de atividade *online,* seguindo as premissas do Interacionismo Simbólico^(11)^, apresentados nos Quadros 2 e 3.

É importante ressaltar que, durante a prática da *Shantala,* o lactente participa da terapêutica como sujeito da ação, e não apenas como passivo no cuidado. Sendo assim, cada criança tem suas preferências e incômodos, que devem ser respeitados, pois, afinal, o objetivo da massagem é o relaxamento, não o estresse do lactente com práticas que não são bem aceitas. Encontra-se com essa interação uma oportunidade de vinculação entre mãe e filho, aprendendo a observar, comunicar-se, entender e respeitar a sua individualidade.

Os estímulos e interações recebidos pela criança são de grande importância para o desenvolvimento cerebral, emocional e intelectual, e têm influência, principalmente, durante o período pré-natal e os três primeiros anos de vida^(17)^. Todos os lactentes que receberam a massagem estavam dentro dos primeiros 1.000 dias de vida, período reconhecido por sua importância e influência nesse desenvolvimento, com impactos a longo prazo^(18)^. Embora a maior ênfase nesse período esteja relacionada à nutrição adequada e ao afeto, outros cuidados devem ser refletidos.

Nesse sentido, o toque físico é marcado como uma possibilidade, sendo essencial para estabelecer a parentalidade positiva entre cuidadores e crianças^(18)^. A interação proporcionada pela *Shantala* pode ser um aspecto que promova o desenvolvimento, especialmente por integrar a necessidade de relacionamentos sustentadores contínuos^(17)^, uma vez que, conforme se davam os encontros, as crianças aceitavam mais a massagem e as mães se mostravam mais confiantes. Com o seguimento da técnica ao longo do crescimento da criança, interações seguras podem ser promovidas.

Sabendo da repercussão que os 1.000 dias terão ao longo da vida, da importância do vínculo e afeto nesse período para a saúde, e considerando que a promoção do bem-estar é uma meta para o desenvolvimento sustentável no mundo, é notório que ações que contribuam para o estabelecimento do vínculo, que visem promover saúde e proteger a saúde materno-infantil sejam difundidas e implementadas, o que demonstra, novamente, a importância deste estudo nesse contexto.

Recomenda-se que, ao final da massagem, seja oferecido o banho à criança, prática que colabora para a continuação do relaxamento^(7)^, uma vez que o banho de imersão em água reduz a resposta do sistema nervoso autônomo, prevalecendo a resposta do sistema nervoso parassimpático. Além disso, é citada a lembrança do ambiente intrauterino como um aspecto subjetivo que pode remeter à sensação de segurança para o lactente^(19)^. A somatória das duas intervenções pode potencializar a interação entre o binômio.

Nesse percurso, atenção especial se voltou à vinculação entre a mãe e o lactente para a promoção do bem-estar conjunto. Todavia, há experiências adversas que podem impactar esse contexto, como os problemas de saúde mental materno. Estudo sobre a ansiedade materna no pós-parto e seus efeitos sobre a amamentação, que também envolve o toque, mostra alta prevalência do transtorno, com pensamentos intrusivos, sentimento de culpa, tensão muscular, entre outros sintomas que podem dificultar a interação com o lactente e diminuir a satisfação com as funções maternas no puerpério^(20)^.

Outro ponto a ser refletido é que a condução dos encontros ocorreu durante a pandemia de COVID-19. Apesar de nenhuma participante ter referido a influência desse cenário nas suas vivências na maternagem, sabe-se que, de forma intrínseca, os impactos das medidas de distanciamento social podem influenciar a saúde mental materna e sua vinculação com o filho. Isso foi visto em estudo brasileiro, no qual observou-se alta prevalência de depressão pós-parto em mulheres durante a pandemia de COVID-19, e esse desfecho esteve associado ao vínculo prejudicado com seu filho^(21)^.

Neste estudo, além das mudanças na percepção materna acerca do bem-estar infantil após a prática da *Shantala,* as mães relataram modificações nas percepções acerca do seu próprio bem-estar conforme a prática da massagem foi sendo aprendida e praticada, como visto na Figura 2, que mostra a transição das sensações “ansiosa”, “sem_dormir” e “esgotada” para “conectada” “calma” e “reduz_ansiedade”. Dessa maneira, cabe refletir em estudos futuros a potencialidade da intervenção para a redução dos problemas de saúde mental supracitados, considerando essa transição para uma maternagem onde respiros são possíveis, como visto no Quadro 3.

Essa maior conexão e vínculo com os filhos, relatada inclusive por aquelas que sentiam-se afastadas da criança, corrobora achados da literatura que descrevem essa mudança na relação com os filhos^(22)^. O vínculo facilita a percepção das preferências do lactente durante a massagem, permitindo dedicar tempo e atenção às partes do corpo onde o lactente expressa sinais de satisfação e a descontinuar a prática aos sinais de inquietação e irritabilidade, sendo um momento no qual as mães podem, também, conhecer e aprender a respeitar as individualidades dos seus filhos^(22)^.

Outro ponto a ser destacado é que a apresentação gradual de técnicas que envolvam estimulação tátil pode facilitar não só a aceitação do lactente, mas também o aprendizado do cuidador, evitando maiores preocupações com o passo-a-passo a ser seguido inicialmente, levando a atenção às reações de seu filho. Em uma perspectiva interacionista, os significados de um fenômeno são tidos após uma reflexão e interpretação^(11)^. Assim, o grupo focal ter sido realizado em cinco momentos diferentes é uma potencialidade deste estudo, pois permite que as percepções das mulheres não sejam imediatas, mas sim relatadas após um processo reflexivo, com mais de uma exposição e estímulo à realização da massagem. Esse tempo colabora para minimizar percepções, como a descrita por um participante do estudo, de autoculpabilização pelo desconforto do bebê ao toque.

O Mapa de Evidências da Efetividade Clínica da Prática de *Shantala,* que reuniu 37 estudos sobre esse recurso terapêutico, demonstrou efeito positivo da técnica sobre o desfecho “bem-estar, qualidade de vida e vitalidade”, com impacto sobre o crescimento, estresse, desenvolvimento neuropsicomotor, qualidade do sono e melhora na propriocepção^(23)^, o que corrobora as percepções maternas visualizadas neste estudo. Ainda, no mapa, não foram encontrados dados referentes ao bem-estar materno^(23)^. Considerando isso, este estudo preocupou-se com as percepções maternas sobre seu bem-estar durante a realização da massagem, demonstrando aspectos positivos e contribuindo no avançar de conhecimento sobre a massagem *Shantala* (Quadro 3).

Nos grupos *online,* as mães davam mais atenção à criança, mesmo estimuladas a contarem sobre sua experiência. Esse aspecto pode estar relacionado ao papel social da mulher como mãe, fenômeno que indica que essa se entrega à função, abdicando da sua individualidade, necessidades e desejos, não podendo se frustrar^(24)^. Nesse sentido, a oportunidade de reunir mulheres vivenciando momentos de vida semelhantes pode ter facilitado a abertura para uma conversa franca, resultando na exposição de seus medos e insatisfações com as funções maternas, mas podendo ressignifica-las após interação com a massagem. Corroborando esses achados, estudo norueguês concluiu que o tempo com a criança na massagem *Shantala* proporcionou alívio da culpa materna relacionada a não sentirem amor constantemente na relação com o filho, o que as envergonhava^(22)^.

Aqui, focou-se na percepção materna. Todavia, para estudos futuros, recomenda-se a abordagem de outros membros da família para a promoção dos benefícios referidos a todos os envolvidos no processo de cuidar. Há um intenso movimento na prática clínica e na literatura científica para a inclusão da figura paterna nas intervenções^(25)^. Neste estudo, uma mãe referiu que ensinou a técnica ao pai da criança, aspecto positivo e que deve ser reproduzido em estudos futuros, para a garantia da parentalidade positiva entre a díade (mãe e pai).

Outro aspecto é que as mães perceberam a configuração da intervenção sendo *online* como algo positivo e promotor da sua participação. Após a pandemia de COVID-19, os profissionais iniciaram sua aproximação com o cuidado remoto^(26)^, e este estudo demonstra que essa organização é promissora para o ensino de intervenções de cuidado. Ainda, recomenda-se para estudos futuros o teste da intervenção em locais como atenção primária (consultas de puericultura e grupos de pré-natal) e serviços terciários (maternidades e unidades de cuidados neonatais), de forma a possibilitar que os efeitos aqui citados sejam vivenciados por crianças e mães em diferentes contextos.

À luz interacionista, os significados surgem a partir da interação que o ser humano tem com outros^(11)^. Pode-se considerar que um fator importante para esse processo, neste estudo, esteja relacionado às atividades educativas realizadas coletivamente, corroborando os achados de estudo sobre a abordagem de cuidado em grupo para a saúde materno-infantil, desenvolvido em um país da América Central, que demonstrou que as atividades coletivas de cuidados permitiram aos participantes fazerem mudanças positivas no comportamento de saúde para si e seus filhos^(27)^.

A transição para a maternidade engloba mudanças fisiológicas (hormonais e neurobiológicas) e no contexto social e ambiental, podendo ocasionar impactos cognitivos, com reflexos ainda incertos e desconhecidos até o envelhecimento. Aqui, o que cabe discutir é que a carga cognitiva que a parentalidade impõe ao cuidador, majoritariamente uma mãe, é tão intensa que exige atenção e cuidado contínuo^(28)^. Nesse sentido, assim como para o bem-estar infantil, é primordial refletir em estratégias de promoção de bem-estar e saúde materno para cumprir a meta da Agenda 2030. A *Shantala* pode ser um recurso simples, acessível e potente para esse fim. Vale aqui destacar a importância que os profissionais de saúde, especialmente os enfermeiros, têm para a realização dos ODS, direcionando ações que promovam a saúde e o bem-estar^(29)^.

Reflete-se que, no cenário brasileiro, apesar de as PICS serem reconhecidas em política desde 2006^(6)^, a sua inserção na prática profissional está ocorrendo de forma gradual. Para que esse processo se acelere e as práticas sejam reconhecidas como tecnologias de cuidado, é necessário a aceitação dos profissionais de saúde, sendo a difusão dos benefícios da prática em estudos um pontapé inicial para um processo de implementação. Destaca-se o papel do enfermeiro nesse processo, como visto neste estudo, onde profissionais da área possibilitaram o aprendizado da massagem *Shantala* por meio *online,* propiciando benefícios no bem-estar materno-infantil, corroborando a literatura, que ressalta a importância desse profissional na difusão das PICS^(30)^.

### Limitações do estudo

Este estudo apresenta como limitações o número de participantes, contudo isso pode ser um reflexo das percepções vistas na subcategoria 1.1, na qual as mães tinham o receio de iniciar a massagem pelo comportamento do lactente quanto ao toque. Esse aspecto reforça a necessidade da publicação deste manuscrito para ressignificar este contexto. A abordagem *online,* apesar da validação materna como um potencial para a sua participação, pode ter limitado a exposição das percepções, além da ausência de controle, se as mães conduziam as massagens diariamente, apesar de envios diários de lembretes, o que poderia influenciar as percepções. Outra limitação refere-se a todas as mães serem residentes de um único estado brasileiro, recomendando-se que estudos futuros atuem em outras estados e países.

### Contribuições para a área da enfermagem, saúde e políticas públicas

Espera-se que este estudo contribua para a enfermagem a partir da validação materna de uma tecnologia de cuidado (massagem *Shantala)* que deve ser difundida dentro do escopo profissional por seus efeitos no bem-estar materno infantil, avançando no caminhar para atingir o 3° ODS.

## CONSIDERAÇÕES FINAIS

Observou-se que as mães perceberam a realização da massagem *Shantala* como um cuidado que promove impactos mútuos no bem-estar materno-infantil, sendo condizentes com a proposta do terceiro ODS e permitindo um caminhar para atingi-lo. Inicialmente, as mães apresentaram receio da realização da massagem pelo comportamento diário do filho e relataram uma exaustão com a maternagem. Todavia, na sua interação com a massagem *Shantala,* essas percepções foram ressignificadas.
